# Supplementary material for: Single Cell Transcriptomic and Chromatin Profiles Suggest Layer Vb Is the Only Layer With Shared Excitatory Cell Types in the Medial and Lateral Entorhinal Cortex
Source: Front Neural Circuits. 2022 Jan 26;15:806154. doi: 10.3389/fncir.2021.806154 (PMC8826650; doi:10.3389/fncir.2021.806154)
Supplement: Supplementary file 14 [file Data_Sheet_1.ZIP › Exc_vs_Inh_Motifs/homerResults/motif5.similar.html]

motif5

## Information for motif5

C
T
G
A
A
T
G
C
T
G
C
A
A
G
C
T
C
G
T
A
A
C
G
T
C
T
G
A
A
C
G
T
  
Reverse Opposite:  

G
T
C
A
A
G
C
T
C
G
T
A
A
C
G
T
C
T
G
A
A
C
G
T
T
A
C
G
G
A
C
T
  

|  |  |
| --- | --- |
| p-value: | 1e-350 |
| log p-value: | -8.077e+02 |
| Information Content per bp: | 1.712 |
| Number of Target Sequences with motif | 24988.0 |
| Percentage of Target Sequences with motif | 59.24% |
| Number of Background Sequences with motif | 5381.9 |
| Percentage of Background Sequences with motif | 49.51% |
| Average Position of motif in Targets | 359.6 +/- 250.6bp |
| Average Position of motif in Background | 338.9 +/- 289.0bp |
| Strand Bias (log2 ratio + to - strand density) | 0.0 |
| Multiplicity (# of sites on avg that occur together) | 1.69 |
| Motif File: | file (matrix) reverse opposite |

### Similar de novo motifs found

|  |  |  |  |  |  |  |  |
| --- | --- | --- | --- | --- | --- | --- | --- |
| Rank | Match Score | Redundant Motif | P-value | log P-value | % of Targets | % of Background | Motif file |
| 1 | 0.643 | A C T G A C T G A C G T C G T A A C G T C G T A A C G T A C T G A G T C A G T C | 1e-341 | -785.819046 | 71.65% | 62.53% | motif file (matrix) |
| 2 | 0.611 | C G A T A C G T C G T A A G C T C T G A C G A T C G T A C G T A A C G T G C T A | 1e-292 | -674.147734 | 20.01% | 13.56% | motif file (matrix) |
| 3 | 0.653 | C A G T C A G T C A T G A C T G C G T A G T A C C G T A G C A T C G T A C A G T G A T C G C A T | 1e-130 | -301.628914 | 54.58% | 48.64% | motif file (matrix) |
